# Supplementary material for: Gender Differences in Dietary Patterns and Eating Behaviours in Individuals with Obesity
Source: Nutrients. 2024 Dec 6;16(23):4226. doi: 10.3390/nu16234226 (PMC12121239; doi:10.3390/nu16234226)
Supplement: Supplementary file 1 [file nutrients-16-04226-s001.zip › nutrients-3355068-supplementary.pdf]

nutrients

Copyright: © 2024 by the authors. Licensee MDPI, Basel, Switzerland. This article is an open access article distributed under the terms and conditions of the Creative Commons Attribution (CC BY) license (https://creativecommons.org/licenses/by/4.0/).

Table S1. Typical Responses to Dietary Behavior Questions by PCA Group.

PCA	Time of Day When Hungry	Do You Ever Miss Meals?	Do You Eat Distracted or Not at the Table?	Do You Eat Quickly?	Do You Eat Alone or Together?	Do You Eat Uncontrollably When Not Hungry?	Do You Wake Up to Eat at Night?
Disordered/Impulsive Eaters	Before Dinner	Yes	Yes	Yes	Often Alone	Often (>1/week)	Rarely / Yes
Irregular Eaters	Before Dinner	Yes	Yes	Yes	Often Alone	Rarely (once a month)	No
Social Eaters	Before Dinner	No	No	Yes	Often Together	No	No
Structured Eaters	During the morning	No	No	No	Often Together	No	No

This table presents the typical responses to dietary behaviour questions used to categorise patients into four PCA groups: Disordered/Impulsive Eaters, Irregular Eaters, Social Eaters, and Structured Eaters. Responses include patterns related to timing of hunger, meal skipping, eating attentiveness, eating speed, social context, uncontrollable eating, and nighttime eating.

Table S2. Confidence Intervals (CI) for Anthropometric and Demographic Variables Across PCA Groups and Genders.

Variable	Disordered/Impulsive Eaters F 95% CI	Disordered/Impulsive Eaters M 95% CI	Disordered/Impulsive Eaters Total 95% CI	Irregular Eaters F 95% CI	Irregular Eaters M 95% CI	Irregular Eaters Total 95% CI	Social Eaters F 95% CI	Social Eaters M 95% CI	Social Eaters Total 95% CI	Structured Eaters F 95% CI	Structured Eaters M 95% CI	Structured Eaters Total 95% CI
**Age**	43.67 to 48.13	42.01 to 47.50	43.71 to 47.17	42.86 to 47.90	38.96 to 43.56	41.43 to 44.85	41.40 to 47.97	38.65 to 44.69	41.03 to 45.54	43.28 to 52.78	43.13 to 50.43	44.40 to 50.16
**Weight**	88.80 to 93.08	103.94 to 110.07	95.31 to 99.37	87.70 to 92.20	104.07 to 110.21	97.08 to 101.51	90.26 to 95.63	99.10 to 106.21	95.11 to 99.77	83.96 to 91.98	99.10 to 105.89	93.64 to 99.73
**BMI**	34.04 to 35.54	33.99 to 35.56	34.23 to 35.33	33.36 to 34.70	34.06 to 35.76	33.95 to 35.06	34.05 to 35.76	33.00 to 34.50	33.79 to 34.95	33.23 to 36.02	31.95 to 33.68	32.76 to 34.32
**AC**	107.44 to 110.62	113.41 to 117.42	110.27 to 112.88	106.65 to 110.02	113.96 to 117.73	111.06 to 113.77	107.13 to 111.04	111.94 to 116.40	109.91 to 112.97	106.19 to 111.75	110.52 to 115.23	109.47 to 113.15
**Fat Mass (kg)**	38.01 to 41.37	32.95 to 37.05	36.49 to 39.15	37.10 to 40.18	33.85 to 37.70	35.81 to 38.35	37.65 to 41.47	31.76 to 36.08	35.44 to 38.45	35.47 to 41.09	29.08 to 33.22	32.16 to 35.84
**Fat Mass (%)**	41.08 to 44.00	28.67 to 32.25	36.38 to 39.08	41.77 to 43.47	31.16 to 35.47	36.21 to 38.92	41.23 to 43.38	30.56 to 33.75	36.34 to 38.87	41.88 to 44.48	28.88 to 31.61	33.69 to 37.16
**FFM**	47.74 to 49.71	66.44 to 69.69	54.96 to 57.90	47.53 to 49.71	66.10 to 69.23	57.46 to 60.49	49.35 to 52.07	63.28 to 67.32	55.77 to 59.18	45.80 to 48.60	65.57 to 70.07	56.85 to 62.29

**Body Water**	36.33 to 37.83	50.24 to 52.72	41.71 to 43.93	36.03 to 37.73	50.26 to 52.44	43.62 to 45.87	37.30 to 39.48	48.35 to 51.31	42.37 to 45.01	34.40 to 36.79	49.22 to 52.19	42.70 to 46.62
**BMR**	1574.61 to 1637.07	2105.82 to 2214.53	1782.26 to 1871.09	1568.82 to 1645.67	2113.80 to 2216.87	1863.56 to 1957.83	1605.79 to 1700.11	2005.56 to 2141.65	1794.21 to 1901.42	1489.46 to 1588.20	2057.43 to 2207.86	1812.70 to 1977.54

This table provides the detailed 95% Confidence Intervals (CI) for anthropometric and demographic variables (Age, Weight, BMI, Fat Mass, Fat Mass (%), AC, FFM, Body Water, BMR) stratified by PCA groups (Disordered/Impulsive Eaters, Irregular Eaters, Social Eaters, and Structured Eaters) and gender (Total, Male, Female). These data complement the summary statistics provided in Table 3 of the main manuscript.

Disclaimer/Publisher's Note: The statements, opinions and data contained in all publications are solely those of the individual author(s) and contributor(s) and not of MDPI and/or the editor(s). MDPI and/or the editor(s) disclaim responsibility for any injury to people or property resulting from any ideas, methods, instructions or products referred to in the content.
